# Hypertension and Cerebral Small Vessel Disease: A Review of the Pathophysiology, Progression, and Prevention

**DOI:** 10.7759/cureus.92760

**Published:** 2025-09-19

**Authors:** Ali Osman, Adel Kanaan, Sami Azeroual, Mohammad Abubakr, Jalal Jwayyed, Rayyan Bhutta

**Affiliations:** 1 Neurology, Ohio University Heritage College of Osteopathic Medicine, Dublin, USA; 2 Cardiology, Marshall University Joan C. Edwards School of Medicine, Huntington, USA; 3 Internal Medicine, Summa Health, Akron, USA; 4 Cardiology, Northeast Ohio Medical University, Rootstown, USA

**Keywords:** cerebral small vessel disease, cognitive decline, hypertension, microvascular ischemia, stroke prevention, white matter lesions

## Abstract

Chronic hypertension often leads to cerebral small vessel disease (CSVD), a condition marked by cumulative damage to the small arteries and arterioles within the brain. Radiologically, CSVD is associated with white matter hyperintensities, lacunar infarcts, and cerebral microbleeds. Clinically, it often presents with cognitive dysfunction, gait abnormalities, and an elevated risk of stroke. Histopathological changes in CSVD due to hypertension include thickening of arteriolar walls, breakdown of the blood-brain barrier, and chronic cerebral ischemia. CSVD is commonly seen in patients with long-standing hypertension, but it is not systematically screened for and is often undiagnosed. Few risk stratification models are incorporated in the outpatient management of hypertensive patients. The primary management strategy for CSVD is stringent control of blood pressure, but the precise target levels and preferred antihypertensive medications to prevent disease progression are not well-defined. Early identification of patients at risk for developing CSVD with ambulatory blood pressure monitoring, cognitive assessment, and brain imaging before the development of functional impairment may be a strategy to consider. In the future, we anticipate a more interdisciplinary care model for CSVD to help delineate the therapeutic window for treatment. Longitudinal studies that evaluate the inclusion of CSVD in the management of hypertension in standard practice will be important to incorporate.

## Introduction and background

CSVD is a neurodegenerative disease of the perforating arterioles, venules, and capillaries that supply the brain parenchyma. It is defined radiographically by a heterogeneous pattern of white matter hyperintensities (WMHs), lacunes, cerebral microbleeds (CMBs), and enlarged perivascular spaces [1,2]. These lesions develop cumulatively and result in a broad spectrum of neurologic sequelae in older adults, including vascular cognitive impairment, gait dysfunction, mood disorders, and recurrent stroke [1]. Chronic hypertension is the best understood and most important modifiable risk factor for CSVD [3]. Chronically elevated blood pressure in arterioles and capillaries causes endothelial dysfunction, lipohyalinosis, and fibrinoid necrosis of the vessel walls. Subsequently, there is hypoperfusion, blood-brain barrier disruption, and chronic white matter ischemia [2,3]. The result of accumulating ischemic and hemorrhagic lesions is a syndrome of executive dysfunction, slowed processing, falls, and late-onset depression [4,5]. Recent studies have started to point to the systemic nature of microvascular injury in CSVD. In a recent meta-analysis of cross-sectional studies, researchers found an association between albuminuria (a marker of renal microvascular disease) and total CSVD burden on MRI, supporting an underlying pathophysiology between cerebrovascular and renal small vessel injury [5]. In quantitative MRI analyses, both greater WMH volume and increased CMB count are strongly and independently associated with incident dementia, recurrent stroke, and reduced mobility in elderly hypertensive cohorts [3,6]. Despite a well-established burden of neurologic morbidity from hypertensive microvascular disease, there are no current clinical practice guidelines for hypertension management that include stratifying patients for CSVD risk or recommend screening. Neuroimaging is often sought only after a patient has manifested neurologic symptoms, yet it is common for CSVD to go clinically silent for years [1]. In its early stages, CSVD can often be dismissed as a normal part of the aging process. Recent data from different patient populations have highlighted the prevalence of microvascular brain injury. In a recent retrospective cohort study of patients with acute respiratory distress syndrome (ARDS), cerebral microbleeds were noted in 20% of patients who underwent MRI during hospitalization, even in the absence of neurologic history-indicating that acute, systemic inflammation and hypoxia may also be drivers of microvascular brain injury [6]. As a whole, these studies demonstrate the possibility of not only underrecognition of CSVD but also of prevention of its consequences. The early course of CSVD is often asymptomatic, even in the setting of widespread parenchymal disease. Yet, given the frequency and significant morbidity of clinical sequelae of hypertensive CSVD, there is an important need for screening for microvascular brain disease in the hypertensive population. In this review, we will discuss the pathophysiology of hypertensive CSVD, current limitations in diagnostic practices, and future directions for screening and prevention.

## Review

Pathophysiology of CSVD in hypertension

Building on the radiologic and systemic implications of CSVD, we next explore the microvascular pathology driving these changes. High blood pressure is a key factor that leads to CSVD, as chronic mechanical stress on small penetrating arterioles results in a characteristic sequence of structural changes. Sustained increases in intraluminal pressure promote progressive smooth-muscle hypertrophy, arteriolosclerosis, and lipohyalinosis. These structural changes narrow the vascular lumen and blunt vasoreactivity, laying the groundwork for ischemia and lacunar infarction [7]. Fibrinoid necrosis further weakens the wall, promoting microhemorrhage in vulnerable regions such as the basal ganglia. The majority of cases are due to sporadic hypertension-related disease, but rare neurogenic locus notch homolog protein 3 (NOTCH3) loss-of-function mutations [cerebral autosomal dominant arteriopathy with subcortical infarcts and leukoencephalopathy (CADASIL)] have shown how intrinsic vascular smooth-muscle disorders can mimic the same radiological and clinical features. However, common NOTCH3 polymorphisms have not been found to confer additional CSVD risk in hypertensive individuals [8]. These arterial changes collectively undermine cerebral autoregulation, resulting in chronic underperfusion of white matter even with modest fluctuations in systemic blood pressure. Early, often subclinical disruption of the blood-brain barrier (BBB) is a critical downstream consequence in this cascade. Endothelial inflammatory signaling, tight-junction protein breakdown, and increased paracellular permeability caused by stretch and shear stress secondary to hypertension result in leakage of leukocytes and plasma proteins into the perivascular space [9].

Furthermore, endothelial injury impairs neurovascular coupling, leading to diminished rapid vasodilatory responses that are important in the usual homeostatic balancing of local blood flow to neuronal activity [10]. Accumulation of microscopic hemorrhages occurs both in deep and lobar regions. In the elderly, this often occurs when cerebral amyloid angiopathy and hypertensive arteriopathy coexist [11]. As a result, MRI-based small-vessel disease scoring systems that take into account lacunes, perivascular space enlargement, white-matter hyperintensity burden, and microbleed topography provide a useful means of gauging the cumulative impact of these processes. 

A feed-forward loop of oxidative stress is established in the setting of endothelial dysfunction and chronic cerebral hypoperfusion. Reactive oxygen species are generated by reduced nitric-oxide bioavailability, increased expression of nicotinamide adenine dinucleotide phosphate (NADPH) oxidases, and mitochondrial dysfunction. Lipids, mitochondrial membranes, and pro-inflammatory transcription pathways are all oxidized by these factors, hastening vascular and parenchymal damage [12]. Experimental and clinical findings support oxidative stress as the common pathophysiological pathway that links hypertension to autoregulatory failure, blood-brain barrier disruption, and white matter loss. As a result, in addition to strict blood pressure control, adjunct antioxidant approaches such as NADPH-oxidase inhibitors or free-radical scavengers such as edaravone should be considered [13].

Additionally, CSVD often smolders for years before clinicians notice it. Population studies show that expansion of periventricular white-matter hyperintensities (WMH) tracks with a measurable slowdown in mental processing speed in otherwise healthy elders [14]. Vascular injury also predisposes to mood disturbance: depressive symptoms rise with increasing WMH, lacunes, and cerebral microbleeds [15]. As damage spreads to fronto-subcortical circuits, motor phenotypes emerge-gait and balance impairment rank as the second most common consequence of CSVD, and bladder urgency often accompanies this gait disorder [14,15]. Because this slow triad of cognitive slowing, depression, and gait-bladder dysfunction looks like “normal aging,” or is mislabeled as primary Alzheimer’s disease when memory loss predominates, many patients remain undiagnosed until disability is entrenched [15,16].

When neuroimaging is finally obtained, the diagnosis becomes obvious: MRI routinely discloses the systolic blood pressure intervention trial-memory and cognition in decreased hypertension (SPRINT-MIND)-defined spectrum of WMH, lacunes, enlarged perivascular spaces, and other lesions [14,16]. Yet brain imaging is still omitted from standard hypertension work-ups because penetrating arterioles are “difficult to observe radiographically,” leaving clinicians with “limited means to assess CSVD prior to the development of clinical sequelae” [14]. Even when lesions are detected incidentally, there is no universally accepted algorithm to stratify neurological risk or tailor treatment intensity in hypertensive patients; the 2021 European Stroke Organisation guideline on covert CSVD concluded that most of the available evidence is “little and low quality,” and therefore stopped short of endorsing specific targets beyond general blood-pressure control [17]. This evidence gap perpetuates under-recognition and under-treatment, exemplifying the need for systematic neuroimaging and prospective trials that link lesion burden to individualized vascular-risk thresholds.

Risk stratification and screening tools

With chronic hypertension being the most significant modifiable risk factor leading to the development of CSVD, our discussion turns to risk stratification and screening tools. As discussed above, the relationship between BP and CSVD is complex. It is not simply an association of isolated elevated BP readings but a chronic change over time leading to the development of white matter hyperintensities, lacunes, microbleeds, and perivascular space enlargement, which then manifest clinically as cognitive impairment and sometimes even stroke [18].

This is why ambulatory blood pressure monitoring (ABPM) has been shown to be superior to in-office monitoring for risk stratification in CSVD. Key markers that can be captured through ABPM include BP variability, diurnal BP, dipping status, and pulse pressure variability [19,20]. These parameters are independently associated with an increase in CSVD burden [21]. Normally, blood pressure should naturally decrease overnight when a person is asleep, reflecting increased parasympathetic drive and a concurrent decrease in sympathetic activation. This is known as nocturnal dipping. Non-dipping and reverse-dipping patterns, where either there is no 10-20% decrease in BP or where there is actually an increase in BP, are linked to increases in the neuroimaging changes discussed above and reflect a higher risk for both incident and progressive CSVD [21,22]. Elevated BP variability is also a finding that is associated with greater total CSVD burden as well as progression of the aforementioned neuroimaging marker [22,23]. The same is found in cases where pulse pressures are persistently elevated, which is indicative of increased arterial stiffness and is also implicated in CSVD progression, especially in older adults [24]. Many of these key findings are impossible to detect by isolated in-office blood pressure readings. This highlights the importance of ABPM in the risk stratification of CSVD.

When it comes to determining risk based on these key imaging findings, two scoring systems exist. The Fazekas score is used to assess the severity of WMH in periventricular as well as deep white matter on MRI [25], and the total CSVD score assigns point values for each key marker on MRI to determine the global burden [26]. Studies have found both scoring systems to be linked to elevated BP readings as well as risk of developing executive dysfunction and global cognitive decline [27]. Despite these findings, there are no established guidelines that recommend routine screening MRI for asymptomatic patients with hypertension [28].

When it comes to the modification of hypertension, BP lowering has been shown to slow further progression of white matter lesions and reduce the risk of mild impairment and dementia in high-risk populations [29]. There is a question whether intensive BP control leads to improved outcomes. The SPRINT-MIND trial set out to see if there was a relationship between intensive BP control, defined as SBP < 120mmHg, and standard control, defined as < 140mmHg. In this study, participants were randomized to each group. The primary endpoint was to see if intensive control decreased the rate of dementia. The conclusion of the study showed that there was no significant difference between the two groups. This study had to be stopped early because the intensive control group showed significant cardiovascular disease and mortality benefits [29,30]. Because the trial stopped early, they were unable to adequately assess long-term effects as they related to cognitive outcomes. While this leaves the door open for further studies, it cannot be argued that well-controlled BPs play a major role in the treatment and prevention of CSVD, and there is more room for further research to be done. A general guideline is that a BP target of less than 130/80 should be aimed for, because hypotension and cerebral hypoperfusion may also result in exacerbation of cognitive decline [30].

In terms of the specific choice of agents, an individualized approach is preferred, although general recommendations still favor the initiation of angiotensin converting enzyme (ACE) inhibitors/angiotensin II receptor blockers (ARBs) and calcium channel blockers (CCBs) [31]. In addition to the potential neuroprotective effects of ACE inhibitors and ARBs, some recent studies have indicated that CCBs may have a unique role in decreasing WMH burden and perivascular space enlargement [32,33]. With these caveats in mind, the general principles of achieving BP control in the outpatient setting still apply, and maintenance and titration of agents should continue for achieving BP goals. Beyond pharmacologic management, lifestyle modifications have been found to effectively reduce BP. The use of interventions such as the dietary approaches to stop hypertension (DASH) diet, sodium restriction, regular exercise, and alcohol/tobacco cessation has all been found to have significant effects [33,34]. These, along with the management of other comorbid conditions like hyperlipidemia, diabetes, obesity, and sleep apnea, all play into the overall management of hypertension and reduction in the development and progression of CSVD [34].

Emerging and adjunctive strategies

As the cumulative burden of cerebral small vessel disease expands within our increasingly comorbid and aging population, multimodal therapeutic approaches are being developed to target underlying pathophysiologic mechanisms, in addition to blood pressure (BP) control alone. Investigational therapies, evolving imaging, and digital monitoring modalities may serve as adjuncts to early diagnosis, risk stratification, and intervention. Of particular interest are sodium-glucose cotransporter (SGLT)2 inhibitors. Genetic proxies for SGLT2 and SGLT1 inhibition were associated with fewer WMH, lacunar infarcts, and favorable diffusion tensor imaging (DTI) metrics in a large Mendelian randomization study. Mediation analysis implicated circulating β-hydroxybutyrate and glucose in this effect [35]. SGLT2 inhibition was also found to reduce reactive oxygen species (ROS) production, preserve blood-brain barrier integrity, and downregulate NF-κB signaling in human endothelial cells [36].

ROS and endothelial dysfunction-induced autoregulatory failure are also the targets of nitric oxide (NO) donors, statins, and free radical scavengers. For example, Edaravone, a free radical scavenger, ameliorated the ROS-driven breakdown in BBB, autoregulation, and ultimately white matter rarefaction. NADPH oxidase inhibitors may blunt this pathway by interfering with ROS generation and represent a promising investigational avenue [37]. The end result of these therapeutic interventions will hopefully translate into a stabilization of microvasculature integrity and resilience against chronic ischemia, hypoperfusion, and microhemorrhage. Mechanistically, these approaches are largely driven by an upstream attack on the early BBB dysfunction. For example, one study in this area showed that vascular stretch and shear stress in the setting of chronic hypertension led to paracellular leakage, perivascular inflammation, and white matter rarefaction [36].

Advanced imaging modalities may also be able to pick up the presence of CSVD before clinical symptoms have developed. For instance, DTI has been shown to be highly sensitive to microstructural white matter damage in preclinical CSVD. Tissue changes were especially pronounced in the external capsule, corona radiata, and superior longitudinal fasciculus (tracts highly associated with executive dysfunction) [38]. By contrast, arterial spin labeling (ASL) perfusion MRI has identified widespread hypoperfusion and delayed arterial transit time in CSVD patients. These neuroimaging metrics were associated with lesion burden and cognitive slowing [39]. In addition to standard structural imaging, positron emission tomography (PET) tracers for β-amyloid and tau pathology have also helped differentiate patients with Alzheimer’s disease (AD) from those with CSVD-related cognitive decline. The presence of amyloid PET positivity, lobar cerebral microbleeds, and cerebral amyloid angiopathy coexists in 20% of older adults. In contrast, hypertensive CSVD tends to lead to microbleeds in deep white matter and gray matter structures. Understanding these mixed pathologies are critical for prognostic and therapeutic reasons [40].

All of these tools are increasingly being harnessed in machine learning pipelines for risk stratification. For example, one study using an imaging-based classifier applied DTI and ASL-derived features to demonstrate that patients with vascular mild cognitive impairment (vMCI) could be accurately identified with over 70% accuracy [38]. Cognitive dysfunction in CSVD can often be quite subtle in presentation, such as slowed processing speed or executive impairment. These symptoms may go undetected on the MMSE, for example. Instead, brief and more domain-specific batteries such as the Oxford Cognitive Screen and the Brief Memory and Executive Test may better capture the CSVD-related cognitive dysfunction [41]. Consider also the use of functional scales for frailty, gait, and continence to better inform management and provide a more holistic evaluation.

Adjunctive management is also being revolutionized through remote blood pressure monitoring and wearable technologies. Given the independent association of BP variability with WMH burden and silent infarcts, continuous ambulatory BP monitoring is becoming an increasingly important preventive strategy [42]. Artificial intelligence models trained on physiological and imaging data may help flag patients at high risk for cognitive decline, falls, or stroke, enabling earlier intervention [42].

Special populations and complex cases

Elderly patients with multimorbidity represent a particularly vulnerable population, requiring careful titration of antihypertensive therapy to avoid cerebral hypoperfusion and falls. Cognitive and functional assessments must be individualized, and BP targets may need to be more permissive in this group [41]. Furthermore, racial disparities in CSVD burden and hypertension control persist due to systemic inequities in access to care, healthcare utilization, and social determinants of health [41]. CSVD often coexists with diabetes, silent strokes, and atrial fibrillation (AF). These conditions increase the risk of cognitive impairment and recurrent stroke. For patients with AF, CSVD presents a therapeutic dilemma due to the elevated risk of cerebral microbleeds with anticoagulation. In such cases, interdisciplinary coordination among neurologists, cardiologists, and geriatricians is essential for balancing stroke prevention with bleeding risk [40].

Future directions and recommendations

Mounting evidence also implicates ambulatory BPV as a novel CSVD predictor. In a large meta-analysis of over 12,000 imaging endpoints, higher systolic and diastolic BPV were each independently associated with greater odds of CSVD (predominantly WML), independent of mean BP [43]. However, BPV is underutilized clinically to date. The seamless integration of wearable ABPM and transient cognitive screening (e.g., MoCA) into primary care provider workflows may help to detect at-risk patients earlier.

Advanced neuroimaging and emerging circulating biomarkers are poised to offer additional support in the preclinical detection of CSVD. Arterial spin labeling (ASL) MRI can detect perfusion deficits and delayed transit time, both of which are linked to lesion burden and cognitive slowing in CSVD patients [39]. Additionally, serum neurofilament light chain (sNfL) is associated with subclinical axonal injury, cognitive slowing, and CSVD burden in non-CSVD radiographic patients [44]. A risk stratification framework integrating the aforementioned factors into a multivariate model may assist in early intervention targeting and more accurately directing the intensity of monitoring. It would incorporate ambulatory blood pressure monitoring (ABPM) to assess blood pressure variability, the Montreal Cognitive Assessment (MoCA) to screen for early executive dysfunction, sNfL as a serum marker of neuronal damage, and structured brain MRI scoring systems for lesion burden and disease trajectory quantification.

Several emerging tools show promise for early identification and risk stratification in CSVD, spanning vascular dynamics, imaging biomarkers, serum-based assays, and cognitive testing. Table 1 summarizes the clinical utility and limitations of key modalities under investigation. 

**Table 1:** Emerging Tools for Early Detection and Risk Stratification of CSVD ABPM: ambulatory blood pressure monitoring; ASL: arterial spin labeling; DTI: diffusion tensor imaging; SNfL: serum neurofilament lightchain; MoCA: Montreal cognitive assessment; MRI: magnetic resonance imaging.

Tool	Domain	What It Measures	Clinical Utility	Limitations
ABPM	Vascular	BP variability, dipping status, pulse pressure [21,42]	Detects hypertension patterns that increase CSVD risk [21,42]	Limited availability; not routinely used in primary care [21,42]
ASL MRI	Perfusion Imaging	Cerebral blood flow, arterial transit time [39]	Identifies subclinical hypoperfusion; correlates with lesion burden [39]	Requires specialized MRI sequences [39]
DTI MRI	Microstructural Imaging	White matter integrity (e.g., FA, MD) [38, 40]	Sensitive to preclinical white matter damage [38, 40]	Research setting; not routinely interpreted Clinically [38, 40]
SNfL	Molecular	Axonal Injury [41, 44]	May detect subclinical damage before radiographic changes [41, 44]	Not yet widely available or validated for CSVD [41, 44]
MoCA	Cognitive	Executive function, attention, memory [14, 41]	Detects early cognitive impairment in CSVD [14, 41]	Requires training to administer; ceiling effects in high-functioning individuals [14, 41]

Translation of the above findings requires the establishment of interventional neurovascular clinics that span the specialties of neurology, geriatrics, cardiology, and primary care to holistically manage patients at risk of CSVD. Such clinics may better coordinate BP optimization, transient cognitive and gait assessment, and medication adherence, all in one place. In addition, randomized controlled trials of neuroprotective medications are an unmet need. Mendelian randomization data suggest that SGLT2 inhibition may have a neuroprotective effect in CSVD, demonstrated by reduced microbleeds and preservation of white matter integrity in diabetic and hypertensive cohorts [35]. Real-world efficacy trials of SGLT2 inhibitors, NO donors, antioxidants, and statins in hypertensive patients with early CSVD are an important next step.

To implement these strategies, there will be a concomitant need to create formal clinical practice guidelines for the screening of CSVD in patients with hypertension. Potential clinical practice guidelines might help to standardize and direct the identification of high risk individuals using established risk factors (i.e. age >65 years, increased blood pressure variability, altered gait, or objective slowing) and would be best applied following multidisciplinary development between major professional societies, for example, the American Heart Association (AHA), American Academy of Neurology (AAN), and American College of Physicians (ACP). A consensus on the appropriate time to start neuroimaging, the role for biomarkers, and the implications for action of subclinical abnormalities would help to operationalize early research into clinical practice and reduce the burden of undiagnosed cerebral microvascular disease.

## Conclusions

Chronic hypertension is a common, often undiagnosed and undertreated cause of the most common form of vascular dementia: cerebral small vessel disease. Radiologic features of CSVD are prevalent and visually apparent on head imaging, but screening tools and risk scores are not yet integrated into the routine care of hypertensive patients, resulting in missed opportunities for early identification and prevention to help avoid the clinical syndrome often referred to as "leakage in the brain." Emerging understanding of the roles of blood pressure variability, microvascular injury, and neurodegeneration is shifting the paradigm of clinical hypertension management to consider targets on drivers of variability in addition to absolute pressure targets. New technologies, including ambulatory blood pressure devices, advanced neuroimaging, serum biomarkers, and wearables, are being explored for earlier detection, risk stratification, and targeted treatment. The rising prevalence of CSVD as our population ages will require streamlining of CSVD-focused assessment into routine clinical workflow and implementation of interdisciplinary prevention strategies to combat the growing burden of vascular cognitive impairment and dementia.
